# The separation of trypanosomes from blood by anion exchange chromatography: From Sheila Lanham’s discovery 50 years ago to a gold standard for sleeping sickness diagnosis

**DOI:** 10.1371/journal.pntd.0007051

**Published:** 2019-02-28

**Authors:** Veerle Lejon, Philippe Büscher, Romaric Nzoumbou-Boko, Géraldine Bossard, Vincent Jamonneau, Bruno Bucheton, Philippe Truc, Jean-Loup Lemesre, Philippe Solano, Philippe Vincendeau

**Affiliations:** 1 Institut de Recherche pour le Développement, UMR INTERTRYP, Université de Montpellier-IRD-CIRAD, Montpellier, France; 2 Department of Biomedical Sciences, Institute of Tropical Medicine, Antwerp, Belgium; 3 University of Bordeaux, CHU Bordeaux, IRD UMR INTERTRYP, Laboratoire de Parasitologie, Bordeaux, France; 4 CIRAD, UMR INTERTRYP, Université de Montpellier, CIRAD, IRD, Montpellier, France; Hunter College, CUNY, UNITED STATES

## Abstract

Human African trypanosomiasis (HAT), or sleeping sickness, is a neglected tropical disease that is fatal if untreated, caused by *Trypanosoma brucei gambiense* and *T*. *brucei rhodesiense*. In its 2012 roadmap, WHO targeted HAT for elimination as a public health problem in 2020 and for zero transmission in 2030. Diagnosis of HAT is a multistep procedure comprising of clinical suspicion, confirmation, and stage determination. Suspects are identified on clinical signs and/or on screening for specific antibodies. Parasitological confirmation of suspects remains mandatory to avoid unnecessary toxic drug administration. The positive predictive value of the antibody detection tests is low. Simple parasite detection techniques, microscopic examination of lymph node aspirate, or stained thick blood films lack sensitivity, whereas in *T*. *brucei gambiense* patients, the number of blood trypanosomes may be very low. Parasite concentration techniques are therefore indispensable. Half a century ago, Sheila Lanham discovered a technique to separate trypanosomes from the blood of infected rodents, based on anion exchange chromatography with diethyl amino ethyl (DEAE) cellulose, a weak anion exchanger. Between pH 6−9, trypanosome surface is less negatively charged than that of blood cells. When blood is poured on top of a DEAE cellulose column, blood cells are retained, whereas parasites pass the column together with the elution buffer. The result is a pure suspension of trypanosomes that retain their morphology and infectivity. Because cell surface charges vary among trypanosome and mammal species, the optimal buffer pH and ionic strength conditions for different combinations of host and trypanosome species were established. Lanham's technique revolutionized the diagnosis of HAT. It is indispensable in the production of the Card Agglutination Test for Trypanosomiasis (CATT), the most used field test for screening in *T*. *brucei gambiense* HAT foci and essential to confirm the diagnosis in suspected people. Lumsden and colleagues developed the mini anion exchange centrifugation technique (mAECT). After adaptation for field conditions, its superior diagnostic and analytical sensitivity compared to another concentration technique was demonstrated. It was recommended as the most sensitive test for demonstrating trypanosomes in human blood. At the beginning of the 21st century, the mAECT was redesigned, allowing examination of a larger volume of blood, up to 0.35 ml with whole blood and up to 10 ml with buffy coat. The plastic collector tube in the new kit is also used for detection of trypanosomes in the cerebrospinal fluid. Unfortunately, mAECT also has some disadvantages, including its price, the need to centrifuge the collector tube, and the fact that it is manufactured on a noncommercial basis at only two research institutes. In conclusion, 50 years after Sheila Lanham's discovery, CATT and mAECT have become essential elements in the elimination of HAT.

Human African trypanosomiasis (HAT), or sleeping sickness, is a neglected tropical disease that is fatal if it remains untreated. It is caused by two closely related parasites: *Trypanosoma brucei gambiense* and *T*. *brucei rhodesiense*. In its 2012 roadmap, WHO targeted HAT for elimination as a public health problem in 2020 and for zero transmission in 2030. Thanks to intensive control efforts, the first objective has nearly been reached

Control and elimination of HAT rely mainly on diagnosis followed by treatment, complemented with tsetse vector control [1]. Diagnosis of HAT is a multistep procedure, comprising of clinical suspicion followed by confirmation of infection and, finally, stage determination. Suspects are identified, based on clinical signs for *T*. *brucei rhodesiense* and/or on screening for specific antibodies for *T*. *brucei gambiense*. Even if treatment options, in particular for *T*. *brucei gambiense*, have improved over the last two decades, parasitological confirmation of suspects remains mandatory to avoid administrating potentially toxic drugs to uninfected persons. Indeed, the positive predictive value of the present antibody detection tests is low, in particular, within a context of elimination in which prevalences below 1 HAT case per 10,000 persons at risk are common. Simple parasite detection techniques, such as microscopic examination of fresh lymph node aspirate or of Giemsa-stained thick blood films are maximally 40% sensitive and have a practical detection limit around 600 to 5,000 trypanosomes/ml, whereas in *T*. *brucei gambiense* patients, the number of trypanosomes in blood may be below 1 to 10/ml [2,3]. Parasite concentration techniques are therefore indispensable.

Half a century ago, Sheila Lanham, working at the Lister Institute of Preventive Medicine in London, discovered a technique to separate trypanosomes, in particular *T*. *brucei brucei*, *T*. *brucei gambiense*, *T*. *brucei rhodesiense*, and *T*. *evansi*, but also *T*. *congolense* and *T*. *vivax*, from the blood of infected mice and rats [4]. Lanham’s technique is based on anion exchange chromatography with di-ethyl amino ethyl (DEAE) cellulose, a weak anion exchanger. Between pH 6 and 9, the cell surface of trypanosomes is less negatively charged than that of erythrocytes, platelets, and leukocytes. As a consequence, when blood is poured on top of a DEAE cellulose column, blood cells are retained, whereas parasites pass the column together with the elution buffer. No platelets and debris are observed. After centrifugation, the result is a pure suspension of viable trypanosomes that retain their morphology and infectivity to mammalian hosts and that constitute the key source for a variety of studies. As cell surface charges vary among trypanosome and mammal species, the optimal buffer pH and ionic strength conditions for different combinations of host and trypanosome species were established [4,5]. Compared to salivarian trypanosomes, like *T*. *brucei*, DEAE is less suited to purify stercorarian trypanosomes like *T*. *cruzi*, the parasite causing Chagas disease, and *T*. *lewisi*, due to their different adherence characteristics [5]. The potential of this technique for diagnostic purposes was evocated by Lanham herself: "*The technique may also be useful in screening potential animal reservoirs of certain trypanosome species and in detecting trypanosomes in Gambian sleeping sickness patients*, *since an unequivocal diagnosis still rests on finding the organism*, *a procedure which can be notoriously difficult in this disease*" [5].

Lanham's technique is seldom used for diagnosis of animal trypanosomosis, but, without any doubt, it revolutionised the diagnosis of HAT. It is indispensable in the production of the antigen (purified trypanosomes) for the Card Agglutination Test for Trypanosomiasis (CATT), a direct agglutination test developed in 1978 and is still the most widely applied field test for screening between 1 and 2 million per year of people living in *T*. *brucei gambiense* HAT foci and is essential for the confirmation of HAT diagnosis [1,6]. Trypanosomes possess a remarkable process of antigenic variation on their surface glycoprotein, and so parasites expressing LiTat 1.3 variant surface glycoprotein (VSG), a predominant variant antigen of *T*. *brucei gambiense*, are purified, fixed, and stained for card agglutination test for trypanosomiasis (CATT)/*T*. *brucei gambiense* production. Lumsden and coworkers developed the mini anion exchange centrifugation technique (mAECT), making use of a flame-sealed glass Pasteur pipette for collection and examination of the column eluate [7]. After adaptation of the mAECT for parasitological diagnosis of HAT under field conditions (Fig 1), its superior diagnostic and analytical sensitivity compared to another concentration technique, the microhaematrocrit centrifugation [8] and to Giemsa stained thick drop examination, was demonstrated [9]. Soon after, mAECT was adopted by WHO and recommended as the most sensitive test for demonstrating trypanosomes in human blood [10].

**Fig 1:**
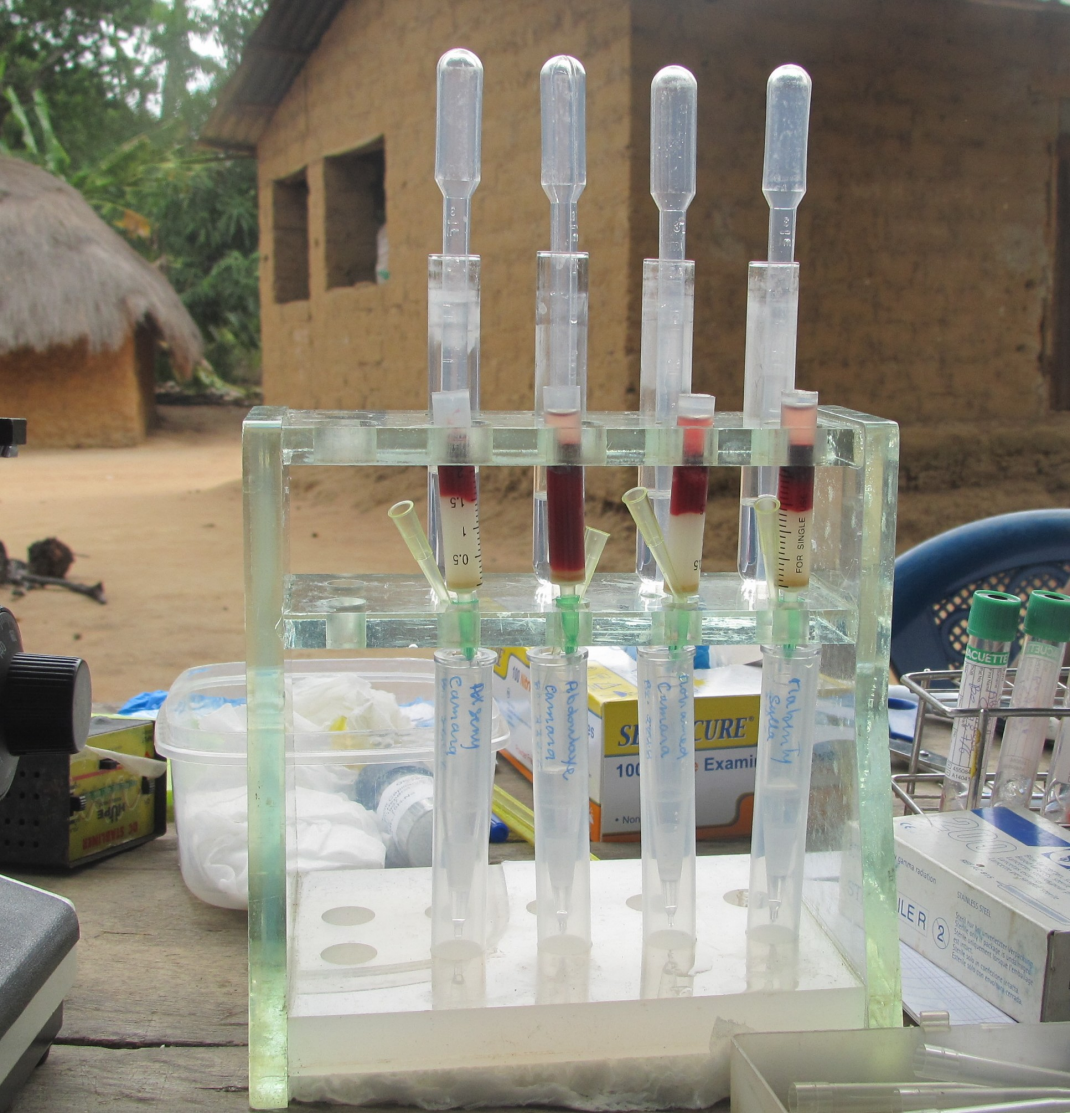
DEAE cellulose minicolumns for the diagnosis of HAT in CATT-positive suspects during mass screening. CATT, Card Agglutination Test for Trypanosomiasis; DEAE, di-ethyl amino ethyl; HAT, human African trypanosomiasis.

With the help of WHO, an mAECT production unit was set up at the Projet de Recherches Cliniques sur la Trypanosomiase (PRCT) in Daloa, Côte d’Ivoire, covering the needs for West-Africa. At the beginning of the 21st century, the mAECT was redesigned, allowing examination of a larger volume of blood, up to 0.35 ml with whole blood and up to 10 ml when buffy coat is applied [11,12]. The analytical sensitivity of this mAECT is less than 50 parasites per ml [3]. The plastic collector tube in the new mAECT kit is also used for detection of trypanosomes in the cerebrospinal fluid [11,13]. In 2007, a second mAECT production unit was established at the Institut National de Recherche Biomédicale (INRB) in Kinshasa. Today, it has a production capacity of 80,000 mAECTs per year to cover the needs of the Democratic Republic of the Congo, where 85% of all HAT cases occur.

So far, superior diagnostic performance of mAECT over all other available techniques for trypanosome detection in blood has been confirmed [2,3,14]: mAECT increases the diagnostic sensitivity of trypanosome detection by 30% to 40% compared with the next best method, microhaematocrit centrifugation, and, as already observed by Lumsden and coworkers, microscopic examination is easier [2,14]. For external quality control, short videos of live microscopic images can be recorded [15], which further reduces the risk of false positive results. This is an important issue in the context of HAT elimination, in which microscopists are less frequently confronted with a positive diagnosis of HAT and, as a consequence, technical experience risks being progressively lost. Microscopic parasite detection cannot be replaced by molecular diagnostic techniques, which have been investigated in recent years (poor reproducibility, unachievable in field conditions).

Unfortunately, mAECT also has some disadvantages, including its price, the need to centrifuge the eluate collector tube, and the fact that it is manufactured on a noncommercial basis at only two research institutes worldwide. Similarly, the CATT is produced at only one research institute (Institute of Tropical Medicine, Antwerp, Belgium). Any interruption in these barely sustainable production systems will have serious consequences, not only on HAT control efforts, but also on individual HAT patients who, without CATT and mAECT, may remain undiagnosed. The VSGs that are used in the CATT are still purified from trypanosomes that are propagated in rats. The two rapid diagnostic tests that are now currently available are still using this source of native VSGs. The replacement of this abundant source of VSG by recombinant VSG or VSG peptides is under investigation.

In conclusion, 50 years after Sheila Lanham's discovery on how to separate trypanosomes from the blood of infected rodents, CATT and mAECT have become essential elements in the elimination of HAT.

## References

[1] Büscher P, Cecchi, Jamonneau V, Priotto, (2017) Human African trypanosomiasis. Lancet 390, 2397–2409. 10.1016/S0140-6736(17)31510-6 28673422

[2] Lutumba P, Robays J, Miaka C, Kande V, Mumba D, Büscher P (2006) Validité, coût et faisabilité de la mAECT et CTC comme tests de confirmation dans la détection de la trypanosomiase humaine Africaine. Trop. Med. Int. Health 2, 470–478. 10.1111/j.1365-3156.2006.01591.x 16553930

[3] Mumba Ngoyi D, Ali Ekangu R, Mumvemba Kodi MF, Pyana PP, Balharbi F (2014) Performance of parasitological and molecular techniques for the diagnosis and surveillance of *gambiense* sleeping sickness. PLoS Negl Trop Dis 8, e2954 10.1371/journal.pntd.0002954 24921941 PMC4055587

[4] Lanham SM (1968) Separation of trypanosomes from the blood of infected rats and mice by anion-exchangers. Nature 218, 1273–1274. 5656665 10.1038/2181273a0

[5] Lanham SM, Godfrey DG (1970) Isolation of salivarian trypanosomes from man and other mammals using DEAE-cellulose. Exp. Parasitol. 28, 521–534. 4993889 10.1016/0014-4894(70)90120-7

[6] Magnus E, Vervoort T, Van Meirvenne N (1978) A card-agglutination test with stained trypanosomes (C.A.T.T.) for the serological diagnosis of *T*.*b*.*gambiense* trypanosomiasis. Ann. Soc. Belg. Méd. Trop. 58, 169–176. 747425

[7] Lumsden WHR, Kimber CD, Strange M (1977) *Trypanosoma brucei*: detection of low parasitaemias in mice by a miniature anion-exchanger / centrifugation technique. Trans. R. Soc. Trop. Med. Hyg. 71, 421–424. 339422 10.1016/0035-9203(77)90043-8

[8] Woo PTK (1970) The haematocrit centrifuge technique for the diagnosis of African trypanosomiasis. Acta Trop. 27, 384–386. 4396363

[9] Lumsden WHR, Kimber CD, Dukes P, Haller L, Stanghellini A, Duvallet G (1981) Field diagnosis of sleeping sickness in the Ivory Coast. I. Comparison of the miniature anion-exchange / centrifugation technique with other protozoological methods. Trans. R. Soc. Trop. Med. Hyg. 75, 242–250. 6272458 10.1016/0035-9203(81)90326-6

[10] World Health Organization (1983) Trypanosomiasis control manual. African Medical and Research Foundation Nairobi, Kenya, Geneva.

[11] Büscher P, Mumba Ngoyi D, Kaboré J, Lejon V, Robays J, Jamonneau V, et al (2009) Improved models of mini anion exchange centrifugation technique (mAECT) and modified single centrifugation (MSC) for sleeping sickness diagnosis and staging. PLoS Negl Trop Dis 3, e471 10.1371/journal.pntd.0000471 19936296 PMC2775158

[12] Camara M, Camara O, Ilboudo H, Sakande H, Kaboré J, N'Dri L, et al (2010) Sleeping sickness diagnosis: use of buffy coats improves the sensitivity of the mini anion exchange centrifugation test. Trop. Med. Int. Health 15, 796–799. 10.1111/j.1365-3156.2010.02546.x 20497407

[13] Miézan TW, Meda AH, Doua F, Djé NN, Lejon V, Büscher P (2000) Single centrifugation of cerebrospinal fluid in a sealed pasteur pipette for simple, rapid and sensitive detection of trypanosomes. Trans. R. Soc. Trop. Med. Hyg. 94, 293 10975002 10.1016/s0035-9203(00)90327-4

[14] Lumbala C, Bieler S, Kayembe S, Makabuza J, Ongarello S, Ndung'u JM (2018) Prospective evaluation of a rapid diagnostic test for *Trypanosoma brucei gambiense* infection developed using recombinant antigens. PLoS Negl Trop Dis 12, e0006386 10.1371/journal.pntd.0006386 29590116 PMC5898764

[15] Hasker E, Kwete J, Inocencio Da Luz R, Mpanya A, Bebronne, Makabuza J, et al (2018) Innovative digital technologies for quality assurance of diagnosis of Human African Trypanosomiasis. PLoS Negl. Trop Dis 2, e0006664. 10.1371/journal.pntd.0006664 PMC6136689 30212459

